# Exploring data trends and providers’ insights on measles immunization uptake in south-west Nigeria

**DOI:** 10.11604/pamj.2023.46.28.37572

**Published:** 2023-09-19

**Authors:** Marcus Ilesanmi, Babatunde Olujobi, Oluwapelumi Ilesanmi, Valerie Umaefulam

**Affiliations:** 1 Department of Community Health and Epidemiology, University of Saskatchewan, Saskatoon, Saskatchewan, Canada,; 2 Ekiti State Primary Health Care Development Agency, Ado Ekiti, Ekiti State, Nigeria,; 3 Faculty of Health Sciences, Queens University, Kingston, Ontario, Canada,; 4 Department of Medicine, University of Calgary, Calgary, Alberta, Canada

**Keywords:** Measles, immunization barriers, provider’s perspective, Nigeria

## Abstract

**Introduction:**

measles outbreak remains a recurring episode and continues to be responsible for millions of deaths globally every year. This study examines measles immunization coverage and uncovers barriers and enablers to effective provision and uptake of measles immunization services from the supply end and provider´s perspective in a developing nation´s context.

**Methods:**

the study employed a mixed-method approach to explore trends and patterns of measles immunization uptake in Ekiti State-a state in the southwestern region of Nigeria-utilizing DHIS 2014 - 2019 data of 789,518 under 1-year children and complemented the quantitative study with key informant interviews from appointed Immunization Officers in the state. Using deductive methods, we thematically analyzed the interview data using NVivo version 12 while STATA 16 was used to analyze the quantitative data.

**Results:**

the annualized measles immunization coverage ranged between 49% and 86% from 2014 to 2019, which is below the WHO set threshold for measles infection prevention. Caregiver, geographical, human, and infrastructural factors were elicited as barriers, while potential enablers include increased public engagement and enhanced media involvement.

**Conclusion:**

while programmatic efforts are being improved nationally to drive up the uptake, this study provides baseline information for benchmarking the subsequent level of efforts and recommends improved collaboration across contextually similar states to promote program efficiency. The results can inform policy and program development, execution and direct future research on measles immunization to address uptake challenges at both local and central administration levels, especially in the aspect of surveillance and monitoring.

## Introduction

Immunization is the most successful public health measure to date [1,2] enabling prevention of disease at the population level. Approximately two to three million deaths are prevented globally each year through immunization [2,3]. About 23.2 million deaths were prevented by the measles vaccine between 2000 and 2018, resulting in a 73% drop in measles cases globally within that period [4]. Despite these advances, developing countries continue to suffer from several endemic diseases, some of which are vaccine-preventable diseases (VPD). Immunization, therefore, remains a key intervention towards the achievement of the third Sustainable Development Goal (SDG 3) of the United Nations. Among several infectious diseases, measles has received prominent attention internationally due to its high infectivity rate [5,6] and its attendant morbidity and mortality. The World Health Organization (WHO) recommends a 92-95% herd immunity threshold (HIT) for a potential spread of measles to die out should there be a source of measles infection in a particular population [5,7]. While sporadic isolated cases of measles portend some danger to the infected individual, the greatest threat is the possibility of spread to susceptible population groups, especially the non-immune, in places with low coverage rates [8], especially in resource-constrained settings.

The measles immunization program is implemented as an integral part of national vaccine-preventable disease (VPD) programs in Nigerian states. Numerous factors operating at various levels influence vaccination uptake and health outcomes. Many developing nations, including Nigeria, implement immunization activities predominantly through their lowest level of government. Since the late 1980s, immunization programs in Nigeria have been carried out by the Primary Health Care (PHC) system managed by the local government areas (LGAs) [9]. At this level of healthcare delivery, immunization of children is achieved through routine immunization and catch-up supplemental immunization activities (SIAs). Similar to practice in many parts of the world, policy responses have been to align practices with the WHO recommendation of a 2-dose vaccination, although with some level of variance, among countries and subregions [10]. The spaced 2-dose approach is believed to offer adequate protection against future infection from measles virus exposure [10]. Nigeria introduced a dose regimen of measles vaccination (MCV1) in 1978 for children aged 9 months as part of the routine immunization activities [11] and introduced the second measles dose in compliance with the WHO recommendation in 2019 [12].

Disparities in coverage are well known to be associated with geographical locations in Nigeria, wherein urban regions often have better immunization coverage compared with rural locations. This is due to access-related problems, acceptability of services, competing priorities, and outreach limitations [13]. Although a larger proportion of Nigeria has not attained the immunization target of 90% nationally and at least 80% coverage in all districts as set out by the Global Vaccine Action Plan (GVAP), some areas in the southern parts of Nigeria have shown successes in achieving the targets [14]. While there has been a slow progressive increase in overall vaccination coverage in Nigeria across the years; Ekiti State, one of the 36 States has one of the highest immunization coverage rates in the country and the highest for measles coverage at 85.7% in 2013 [15]. Internationally, research into how widespread challenges in childhood measles immunization uptake exists, but very little is known from the context of Ekiti State, southwest Nigeria. With 131,732 measles cases recorded between January 2012 and September 2016 and the resulting death of 817 cases [16], measles seems to still be prevalent in Nigeria. There were 12 confirmed measles cases in Ekiti between 2007 and 2012, which is about 1.5% of all cases in South West Nigeria [17] where Ekiti is 8.5% of the total population at 3.27 million inhabitants [18].

A good understanding of measles vaccination performance in Ekiti State and the exploration of the underlying barriers and enablers to adequate immunization in the state can provide useful baseline information to inform policy, provide a platform for re-designing and modifications to implementation strategies for equitable immunization coverage in this jurisdiction and other contextually similar parts of the country. The aim of this research, hence, was to examine the coverage among Ekiti State Local Government Areas (LGA) and uncover enablers and barriers to effective provision and uptake of measles immunization services at a subregional level to elicit interventions to improve equitable measles' immunization coverage rates that can be used as a benchmark across the nation.

## Methods

Ekiti State, one of the thirty-six states in Nigeria, apart from the Federal Capital Territory is located in the south-west part of the country, with a population of 3,439,134 (2018 projection and 2.3% annual growth rate). The State is administered through 16 LGAs and 177 wards, with at least one health facility in each of the 174 of the 177 wards of the state. Depending on the size and the location, some wards have more than one health facility, a total of 326 primary health centers, 22 secondary health facilities, and two government referral teaching hospitals. Immunization data in the state is collected by Local Immunization Officers (LIOs) and Health Management Information System (HMIS) Officers appointed for the LGA Primary Health Care Departments. They coordinate the collection of data in their respective LGAs and upload the data directly to the District Health Information Software (DHIS) 2.0 platform, where they are accessed at the State and Federal levels for program planning purposes.

Numerous factors operating at various levels influence health, hence a robust framework is essential to systematically address the multiple determinants of health problems where change is desired. We used the health behavior framework [19] to guide this study and to inform its data collection instrument development. The framework integrates constructs of Social Cognitive Theory on self-regulation and group behavior, as well as the interrelationship of Social Learning Theory and the Health Belief Model [20], which have been demonstrated in understanding, predicting, and influencing behaviors. We utilized both quantitative and qualitative data to examine the study objectives. The quantitative strand analyzed a cross-sectional total sample of 789,518 under 1-year children on the DHIS platform from 2014 to 2019 of the 16 LGAs of Ekiti State. We calculated the coverage rate by dividing the absolute numbers of immunized subjects by the target populations, and the result was expressed as a percentage to arrive at the yearly and monthly state-level measles immunization coverage. The qualitative part of the study is grounded in phenomenology due to the shared experience of participants. Interviews were used to collect data from the Government Immunization Officers in Ekiti State as key informants. The study focused on the perspectives of the immunization providers in the service of Ekiti State, which included policymakers and frontline immunization program delivery staff.

All 16 LGAs immunization officers were invited to participate voluntarily as key informants. The participants were identified by the research team, informed of the study via email, and purposefully selected based on their strategic roles in the immunisation policy interpretation and program implementation at the LGAs and the State. These key informants because of their level of experience and direct involvement in the program were believed to have the most in-depth and accurate assessment and expert opinion of the program availability in their respective LGAs. The interviews followed a consistent structure using an interview guide (Annex 1) for approximately 45 minutes via telephone. The participants were allowed to select the appointment and time of the interview to maximize their comfort. Valerie Umaefulam, a qualitative researcher, conducted the interviews. The interviewer had no prior relationship with the participants and was not connected to immunization activities in Ekiti State. Only the research team was present at the interviews and was involved in taking the interview notes. Interview data was collected until data saturation was reached, i.e. no new information was being provided. The interviews were recorded, transcribed verbatim, and the data was analyzed using deductive thematic analysis with NVivo version 12 qualitative analysis software [21]. Two research team members coded and interpreted the data. Participants provided written and verbal informed consent.

**Ethical approval:** the ethical approval reference number EK/PHCDA/ADM/316/37 was obtained from the State Primary Health Care Development Agency, Ado Ekiti, Ekiti State. Also, informed consent was obtained from all the participants during the interviews.

## Results

**Figure 1:**
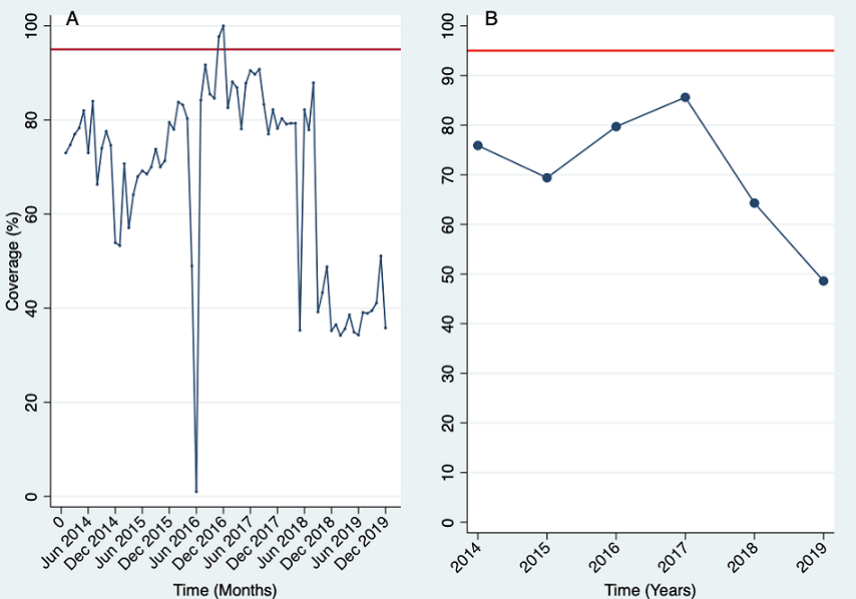
Ekiti State measles immunization coverage (2014 - 2019): A) monthly; B) annualized

**Figure 2:**
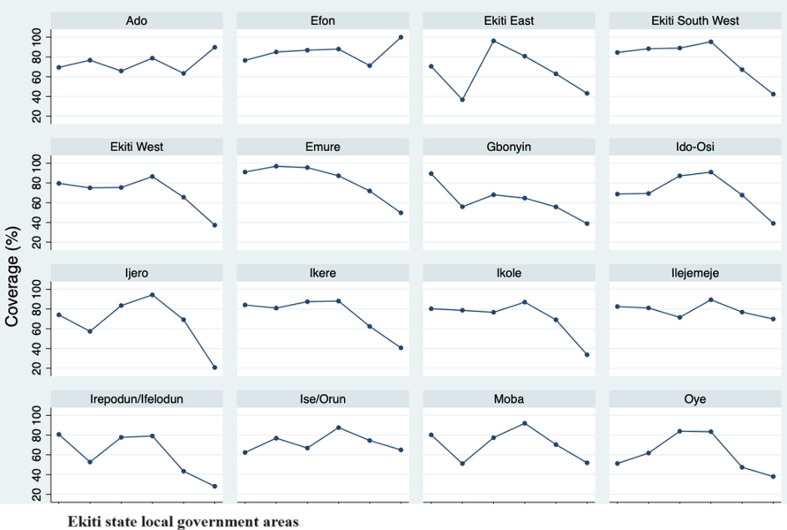
Ekiti State LGAs yearly measles’ immunization <1 year coverage rate (2014 - 2019)

The monthly measles immunization coverage rate from January 2014 to December 2019 in Ekiti State was below the herd immunity threshold (95% and above coverage) between 2014 and 2019 apart from a period of 4 months (November 2016 - February 2017) where there was at least 95% coverage rate (Figure 1A). From August 2018 to December 2019, the coverage rates were below 50% (Figure 1B) reflects the annualized state average coverage rate which increased from 76% in 2014 (90,135 of 121,752) to 86% in 2017 (112,878 of 133,429) followed by a progressive decline to 49% by 2019 (54,332 of 141,830 targets), a 35.53% decline over the study period. When these coverage figures were compared with the 95% herd immunity threshold (HIT) reference line, the state did not attain the desirable HIT between 2014 through 2019 (Figure 1A, B). The aggregate of each LGAs coverage is reflected in the state-level result. At a much granular level, an examination of each Local Government Area (LGA) coverage by year revealed varying levels of differences in coverage rates (Figure 2). Apart from Ado Ekiti and Efon LGAs, where coverages have been relatively constant with an upward trend in 2019, even though HIT was not achieved in that period, there was a downward trend in measles immunization coverage in the remaining 14 LGAs of the state. The coverage differential in the respective LGAs ranged from 12.6% in Ilejemeje LGA to 53.3% in Ijero LGA (Figure 1A, B).

Twelve participants were interviewed. The twelve interviewed participants were state government-appointed immunization officers, all were female, and they had between one to seven years (average of four years) of on-the-job experience. The participants’ responses substantiated the researchers´ understanding of the observed coverage patterns and we summarise the themes and findings from the interviews under three major headings as follows: disparities in immunization coverage, barriers and enablers to achieving herd immunity threshold, and policy interventions and innovations for improved coverage.

**Disparities in coverage:** across the LGAs, there were varying perceptions of immunization coverage. Some LGAs reported successes in coverage, however, the general participant responses indicated a misalignment between targeted and ongoing coverage. A participant stated, *“We are trying out our best, it is improving (P2)”* without reference to the target figure. The disparity in immunization coverage could be due to numerous factors depending on the geographical location of the LGAs. For instance, some communities are limited by the availability of infrastructure such as cold chain equipment to assist immunization service provision, *“The local government is very big and with just one solar-powered NPI unit, we need it in about 4 or 5 axes in this LGA, so that it will be supplying all other axes in our LGA. (P8).”* Other factors involved health facility availability and access. Case in point, a participant stated, *“We still have challenges of health facility in this area, there are some wards without health facilities in which local government rented apartment for us; but, as the government changes, they have not been paying rent, so those wards have been left with no health facilities (P7).”* It was gathered that disparities were contextual in nature.

**Barriers and enablers to measles immunization uptake:** this theme speaks to the reported facilitators and the challenges that influence measles immunization coverage in Ekiti State. While the existing enablers were discussed under ´public engagement´ and the ´positive role of media´, the barriers were identified and classified under ´caregiver and population factors´, ´geographical characteristics´, and ´resource issues (human and infrastructure) ´.

### Enablers

Participants reported that the current SIA program is a good strategy to improve immunization coverage among target age groups. Other ongoing practices at different LGAs promote immunization coverage which includes the provision and maintenance of solar panels, providing immunization reminders to caregivers, use of community mobilizers to identify defaulters, and providing regular immunization sessions to caregivers. Home visits have assisted in boosting immunization and in identifying defaulters, as stated, *“We also tell the health workers to go for home visits to follow up with the LGA routine immunization officers (R1) efforts” and “this has been going on for a while now, ...from there, they can be able to check for settlements to ensure those settlements are well covered in terms of measles immunization (P3)”*. Evening outreach services to community members target farmers and individuals not accessible at daytime impacted coverage, *“We used to have one outreach service per month but now we have increased it to 2 or 3 per month in some wards (P9)”*. The present cooperation, dedication, and willingness to work exhibited by the available health care staff advance immunization coverage in the state. Participants reported that teamwork and integration of immunization services with other ongoing programs have assisted in increasing the reach and uptake, *“Number one is teamwork, then integration with other program officers like malaria, HIV/AIDS, family planning, when they are bringing out their commodities, we use that opportunity to provide immunization services, so we can build on each other for PHC services (P12)”*.

All participants noted that engaging with the public and utilizing community resources to create awareness for immunization activities improved uptake and coverage. For example, a participant mentioned, *“In each ward, we have a social mobilization committee, we do have monthly meeting whereby we talk on the issue of routine immunization with this mobilization committee, even though we live within the community, they [mobilization committee] know the community more than we health workers do... the town announcer moves to canvass for people each time we are having immunization day, they move around to enlighten the people (P7)”*. The engagement activities for advocacy and education involved the local government authorities, the community chiefs, and local health workers. A participant stated, *“We went to visit a palace where all the Kabiyesis [chiefs] in the local government were gathered, we let them know the importance of the immunization in the local government, they involve the community for advocacy and educate them; and... there was an improvement of immunization coverage in such communities (P9)”*. The media also plays a significant role in increasing immunization uptake by supporting mobilization and sensitization activities. The media collaborates with health educators to create TV and radio jingles for the dissemination of immunization information. As stated by a participant, *“the media is going a long way in ensuring higher measles immunization coverage(P3)”*. Nonetheless, media coverage is hindered by limited electricity power supply in some communities to listen to awareness campaigns from the providers, as well as the cost to air immunization messages, *“They [media] will accommodate us while we do whatever we want to do but it is not free (P12)”*.

### Barriers

**Caregiver and population factors:** communication gap between caregivers and health workers creates an obstacle to immunization uptake, *“if the health workers do not inform the mother about when to come back for the next appointment, the mother will not come because she is not aware (P1).”* Other caregiver factors included level of knowledge, perception, experience, and attitudes regarding immunization, all of which create barriers. For instance, a participant noted that *“there might be previous adverse effects following immunization that their child might have experienced, so this may cause this barrier, they may not bring their child to collect (sic) vaccines again (P4).”* Also, low level of awareness and understanding of immunization and age group requirements by caregivers hinder uptake, for example, a participant stated, *“there are some mothers that when you meet them and tell them the essence of immunization, they would tell you, “I´m not even aware that immunization is still being given to 9 months...when my baby got to 2 or 3, I just stopped (P3).”* Culture and beliefs have a significant impact on immunization uptake. Some tribes and religious groups do not receive medication, while some reject vaccination. Case in point, *“Because of their beliefs and culture, most find it difficult to bring their children out for vaccination (P6).”* Also, the nomadic and migrant populations were cited to possess beliefs that affect immunization uptake and coverage. A participant stated, *“People refused to take even Oral Polio Vaccine (OPV); even what is not immunization, they rejected it, they didn´t take it (P11).”*

**Geographical characteristics:** distance affects measles immunization coverage within Ekiti state, especially access to and from health facility locations in some communities. A participant indicated, *“Most of the time, the challenge is the location or distance of the health facilities which may be too far from the communities, especially the model health centers that are put at the end of each community; some people may not be able to get there (P4).”* Security recently became a concern as health workers find it unsafe to go to some settlements due to ongoing security situations in some parts of the country resulting in fear of working in remote areas *“We have security compromise in this LGA, and we cannot reach many of the outreaches that we are supposed to go (P8)”*.

**Resource issues (human and infrastructure):** all participants reported limited resources as inhibiting immunization service delivery and uptake. This includes the limited technical input from nurses, community health extension workers, and health educators for mobilization purposes. Also, due to limited training and in-service capacity building that are far between, there is a knowledge gap among health workers. All participants reported the imbalanced distribution of trained staff in LGAs, particularly in the rural areas. *“...uneven distribution of the staff is a problem because we don´t have enough community health extension workers whose work is specifically immunization (P3)”*. Infrastructure resources were also reported to be a key barrier to immunization coverage. In some instances, either this was absent, or it required an upgrade or replacement. Such infrastructure included healthcare center buildings, cold chain equipment such as solar freezers, fridges, and vaccine carriers. A participant stated, *“We have poor cold-chain maintenance, some equipment is damaged and needs replacement (P5).”* There is inadequate support to carry out immunization outreach sessions, especially in difficult terrains and hard-to-reach locations. The paucity of funds to complement programs like outreach services, community mobilization, ad-hoc staff remunerations, electricity supply, internet data bundle, and transportation to health centers impact immunization coverage. A participant stated, *“There must be funding for the RI as lack of funding is a main problem encountered from collecting vaccines from the local government headquarters to the health facilities, for example, most of the health facilities staff are using their money to collect vaccines. At times they may not be able to finance it... (P4)”*. Another mentioned, *“Funds are the problem, like outreaches, we plan for 4 outreaches, but we see them doing 2 or 1 and it lowers the coverage. So, we still need more funds for the RI workers to be going for outreaches (P5)”*. Poor road structures and inability to transport health workers and vaccines to geographically remote locations affect uptake, *“In my LGA, there is a lot of settlements, towns, and villages, so if there is provision of equipment like motorcycles and vans to convey the health workers to those areas, I think that will improve coverage (P10).”*

### Policy interventions and innovations

Participants suggested interventions focused on providing incentives for immunization, mobilization and outreach initiatives, and specific policy interventions. Providing incentives to caregivers, particularly mothers, was indicated as a strategy that could motivate and enhance the uptake of immunization services. These incentives could be in the form of household items that support childcare, such as insecticide-treated nets (ITNs), food items, and sanitary items. Case in point, a participant stated, *“We need to even give mothers incentives to come out, so the local government can provide for that... If we give them incentives, such as detergents for clothing and pampers for their children, they are more likely to come (P1).”* Another participant noted, *“â'¦mothers should be encouraged by providing them with incentives to motivate them when they bring their children so that those who benefit from such will be able to go back to inform others and invite others (P10).”* Mobilization initiatives were also suggested to assist in sensitizing the community. Participants stated the need for regular immunization promotion campaigns to improve immunization coverage, *“The major thing is that if we sensitize a group today, tomorrow we need to sensitize another group that would have arrived... So, we need frequent mobilization and to be visiting on a regular basis (P11).”* Health educators play a significant role as mobilisers in the communities, emphasizing the need for providing support for mobilisation, *“The health educators with which we work together, they need some support, helping them with batteries to mobilise people in settlement. They need the batteries for the megaphones (P3).”* It was suggested that health educators, community leaders, and religious leaders be educated and involved in sensitization and mobilization to foster community ownership, *“...because they will be the ones to disseminate the information to the doorstep of mothers (P1)”*. Another suggestion was that the state government needs to empower the LGAs to carry out social mobilization exercises to improve coverage. These mobilization interventions could be through radio jingles and TV for urban and moderately rural settlements; and for remote settlements with limited power, infrastructure provision and integration of outreach services with sensitization activities can improve immunization uptake. An increase in outreach services was suggested to further improve immunization uptake and services in communities not easily accessible by road. Outreach services would enable *“services to be made available to the community at their doorstep (P2)”*. Providing outreach services would require supportive supervision and funding for infrastructure such as vans and motorbikes to enable unhindered regular outreaches. A participant noted, *“They should support us by financing the outreaches because some of the children are found in the settlements that the fixed posts cannot cover, so we use outreaches to capture them (P11)”*.

Participants suggested that local government policies can support routine immunization by improving on political commitment and providing funding and other resources for monthly immunization, *“It is not only through funds that they can support us, I know funds are a major thing. They should be committed, they should own immunization, they should see it as their own property and support it. For example, if we need cold chain equipment, let them buy it and put it there. Let them put up a standing committee that is strictly for immunization (P12).”* Other participants suggested more stringent regulations to discourage individuals and communities from refusing immunization for their children. For example, a participant stated, *“We have so many of them that are so adamant, a whole settlement, even the traditional leaders...let there be a law. So that the refusals can be reminded that there is a law in place to punish defaulters (P12).”* Some participants also suggested that immunization be incorporated in the school curriculum to increase its reach and uptake, *“the government should make it compulsory for all the private and public schools especially creche (P10)”*. We provide a summary of the main highlights of the interview results in Table 1.

**Table 1:** summary of service providers' perspectives on immunization uptake

Enablers	Barriers	Policy interventions and innovations
Targeted outreach services	Cultural contextual issues	Incentivisation
Program integration	Geographic and security challenges	Improved community engagement
Community collaboration and engagement	Staffing and funding	Stakeholder education
Media involvement		Political support/commitment
		School curriculum integration

## Discussion

In Ekiti State, the analyzed monthly data showed that coverage improved progressively from 2014 to attain a lower level of HIT range (92%) in August 2016 after a drastic drop to almost zero in June 2016 due to health workers´ industrial action. Research shows that there is a decline in the utilization of health services with distance to health services, and this has a crucial impact on equity since individuals living in remote locations are at a greater disadvantage in accessing and receiving immunization services [22,23]. Our study shows that population/caregiver characteristics can either contribute negatively or positively to immunization coverage. Low literacy and education of mothers/caregivers can negatively impact immunization coverage [13,24]. Recall of dates to administer immunization doses hinders immunization uptake, as caregivers often find it challenging to remember the number of vaccine doses their older children have received and the next scheduled dose [14]. For example, a study in another Nigeria southwest state showed that limited knowledge of immunization schedules displayed by carers undesirably influenced immunization utilization [25]. Personal experiences and trust with immunization are often culturally driven. Our study shows that culture, ethnicity, and religion play a significant role in immunization uptake and coverage. Low overall immunization coverage has religious group implications in Nigeria, with higher immunization coverage being present among children of Christian families compared to Muslim families [26,27].

Systemic and institutional factors related to financing and resource availability create barriers to immunization coverage. Although the local government level is responsible for providing immunization services to their communities, they are often limited with the technical, financial, and human resource capacity to implement primary health care services [9]. Several health facilities often lack an adequate number of trained personnel resulting in overburdening the system and health workers´ fatigue and burnout. Thus, addressing and removing obstacles to immunization access in Nigeria will be profound, especially at the local government level [22]. In Nigeria, although immunization is supported by GAVI and other multilateral organizations, the states and local governments are responsible for salaries, in-service training, and capital costs associated with immunization. Other expenditures such as transportation of immunization commodities, and road infrastructure are the shared responsibility of the federal, state, and local governments [22]. The country established the National Emergency Routine Immunization Coordinating Center (NERICC) with the intended outcome of removing barriers to routine immunization services with optimization of the conduct of fixed and outreach services by health workers and the program managers through Optimized Integrated RI Sessions (OIRIS). In like manner, the country, in collaboration with the development partners, put together the Nigeria Strategy for Immunization and PHC System Strengthening (NSIPSS) [28] which is a 10-year strategy for health system strengthening and PHC-level ownership development for sustainable immunization efforts. The NSIPSS, which will run from 2018 to 2028, identified many challenges to immunization and attempted to proffer solutions to many of those challenges. However, the contextualization of those solutions in the different areas of Nigeria with myriads of culture leaves room for the need to understand the peculiarities of individual geopolitical regions, states, LGAs, and communities for more directed implementation.

Despite these barriers, various factors promote and advance immunization coverage in the state. For instance, our study shows the value in engaging with the public and leveraging community resources to create awareness and better knowledge of immunization activities, as well as providing regular immunization sessions to community members. Related studies showed that involving traditional community leaders in immunization programs and engaging with community leaders using culturally appropriate platforms is essential to further immunization coverage due to their influence in the community [25,29]. Our study also shows that the media is instrumental in sensitization and communicating health messages in the states and can significantly affect immunization coverage. However, useful as the platform can be, it can equally introduce inequalities in health information access and diminish the beneficial impact of mass media [30], especially among those without access to electricity supply, radio, or television. Thus, the importance of using community networks and local information systems to complement the dissemination of information is essential. A study in Southern Nigeria also showed that mobilization and engaging the rural community in delivering immunization services boosted coverage [31]. Additionally, our findings demonstrate that activities by the Community Health Extension Workers (CHEWs), including providing immunization reminders to caregivers and assisting in identifying defaulters, advance coverage. It is to be noted that the Community Health Extension Workerss (CHEW) are trained to spend about 75% of their work hours in the communities [32], to drive demand for routine PHC activities and other child survival services. It is believed the presence of the CHEWs in the communities should have an impact on measles and other VPDs uptake, however, this has not been studied.

National and subnational strategies, along with the supplementation of measles immunization with various SIA campaigns have been effective in boosting Nigeria´s immunization coverage levels [14]. Much as the country rolls out strategies to improve immunization coverage, most of the PHC facilities in Nigeria are overburdened with several factors such as inadequate availability of trained healthcare workers coupled with a lopsided and disproportionate distribution of the existing ones, inadequate supportive supervision, and underperforming or non-existent cold chain system. In 2020, the transmission modes of the COVID-19 virus led to the suspension of SIAs and other Mass Vaccination Campaigns (MACs), which had hitherto boosted coverage figures, as the conduct of such mass gathering exercises has the potential to contribute to community transmission of COVID-19 [33]. It is hoped that with the strategic role of COVID-19 vaccination with high coverage of the population, the state will subsequently be able to scale up her routine immunization exercises and conduct SIAs where necessary to boost the coverage figures.

## Conclusion

This study found that despite low measles' coverage between 2014 and 2019 in Ekiti State, southwest Nigeria, the healthcare providers are equipped with knowledge of factors that have worked and can be improved upon; while public engagement and the role of media have been found positive in mitigating the coverage challenges. These factors can provide vital information for collaboration, policy, and program improvement for measles immunization delivery efficiency and may be a springboard to reducing herd immunity challenges and contributing to third SDG achievement in Nigeria. While this research provides added knowledge on practices and interventions to improve immunization uptake in low-resource settings, the results are useful within applicable contexts but within the following limitations. The researchers were unable to interview LIOs from all LGAs, nonetheless, we found the elicited information sufficient to reach saturation. Future research incorporating insight from all LGA representatives could provide more exhaustive information. Since measles vaccination is effective at offering long-life protection against morbidity from the disease, regular coverage evaluation is essential for informed health programs and policy development.

**Recommendations:** we recommend coordinated resource mobilization to provide sustainable immunization services in the state and all stakeholders' engagement for improved ownership of vaccine-preventable disease programming and control. Governments and responsible civil societies should boost security to enable contact tracing and outreach services, while the reminder system should integrate mass media and telephone modes with the existing local methods to drive demand for measles and other immunization services uptake.

### What is known about this topic

- *Immunization officers have a central role in strategic immunization planning and program implementation for improved uptake;*
- *Improving immunization coverage rates has been a challenge in many developing nations*.

### What this study adds

- *This study uncovers emerging issues and factors affecting measles immunization and other vaccine-preventable diseases uptake from the providers´ lenses;*
- *Findings from this study uncover the need for a more granular level (i.e. the local government areas) surveillance and monitoring of immunization services that are separate but coordinated along with the national and state programs;*
- *The study adds to the body of literature on coverage monitoring at small geographical levels*.

## Annex

**Annex 1**: immunization officers interview guide (PDF - 61KB)
